# Shearwaters make efficient navigational decisions, even at very fine scales

**DOI:** 10.1242/jeb.251556

**Published:** 2026-04-30

**Authors:** Lewis Lancaster-Reeves, Sarah Bond, Joe Morford, Katrina Siddiqi-Davies, Joe Wynn, Patrick Lewin, Alana Halpin, Emma Thornton, Barbara Francik, Max Buckley, Tim Guilford, Oliver Padget

**Affiliations:** ^1^ Department of Biology, University of Oxford, Oxford, OX1 3EL, UK; ^2^ Department of Brain and Cognitive Sciences, University of Rochester, Rochester, NY 14609, USA; ^3^ School of Environmental Sciences, University of Liverpool, Liverpool, L3 5DA, UK

**Keywords:** *Puffinus puffinus*, Navigation, Familiarity, Learning, Route learning

## Abstract

Seabirds are superb long-distance navigators, navigating across oceans with remarkable accuracy. Whilst the mechanisms facilitating these movements are increasingly understood, it is unclear how accurately seabirds determine their position on finer scales. Here, we investigated local navigation in the Manx shearwater (*Puffinus puffinus*) by displacing GPS-tracked individuals to sites around their colony island. Because shearwaters normally avoid flying over land, these release sites present a choice of two over-sea island circumnavigation routes that differ in distance. We found that birds preferentially adopted the shorter round-island route, demonstrating that shearwaters are capable of learning route efficiencies even when differences in payoff are very slight in comparison to the distances they routinely travel. We discuss the learning mechanisms that may underpin this navigational efficiency; including how birds might distinguish between routes where the absolute difference in payoff is minimal, but the relative payoff is large.

## Teaser

**Summary:** A fine-scale displacement of Manx shearwaters, a wide-ranging pelagic seabird, reveals that they preferentially adopt shorter homing routes following release at proximal sites, demonstrating their ability to learn route efficiencies even when payoff differences are very slight.

## INTRODUCTION

Manx shearwaters, *Puffinus puffinus*, routinely travel hundreds of kilometres across open ocean in search of food (Guilford et al., 2008). Constrained to return to their breeding colony as central place foragers, they must then be able to navigate effectively back home from these distant sites and have evolved to be extremely proficient at doing so (Matthews, 1953). Whilst long-distance, at-sea navigation is thought to be facilitated by detecting large-scale gradients in environmental cues, including olfactory cues in the case of shearwaters (Gagliardo, 2013; Gagliardo et al., 2013; Holland, 2014; Phillips et al., 2006), it is unclear whether these cues would remain useful at smaller spatial scales (Lohmann and Lohmann, 2006; Mouritsen, 2018; Phillips, 1996; Wallraff, 2004, 2005). It is therefore of interest to determine (a) how effective shearwaters' navigation systems are at local tasks and (b) what is the mechanistic basis for this.

Our current best understanding of local area navigation is seen in the widely used model system of homing pigeons. Within an area of high familiarity, pigeons can refine their routes through repeated experience with the homing task, increasing their navigational efficiency with repeated releases from the same site (Bonadonna et al., 1997; Guilford and Biro, 2014; Meade et al., 2005). Furthermore, it appears that birds can recall these individually refined routes such that when displaced they will preferentially adopt these routes, even in cases where a more efficient alternative is available (Biro et al., 2004, 2006; Meade et al., 2005). This process of route learning and refinement is likely to be underpinned either by associative (‘trial and error’) learning, where animals encode navigational decisions with the reward of reaching home (or some proxy) or via some form of latent learning, i.e. route refinement without direct reinforcement when the navigational task is achieved, as seen in cognitive map models (Guilford and Burt de Perera, 2017; Wang and Hayden, 2021). If route improvements are learnt associatively, then for a route preference to form there should be a perceptible increase in the reward value, the associated positive affective value, of executing a given route over alternatives (Guilford and Burt de Perera, 2017; Kahnt and Tobler, 2017; Pearce, 2008). At finer scales, however, these distinctions in route value may become less salient (perceptible in a learning context), as the improvements offered by routes become less pronounced. Therefore, evidencing such fine-scale route efficiencies, particularly in a long-distance wild model, would give us insight into the navigation performance of this species across scales, and allude to the mechanisms underpinning this.

Here, we examined fine scale route choice in a species celebrated for its long-distance navigation abilities. Shearwaters routinely avoid flying over land, so landmasses (islands and peninsulas) can become navigational obstacles (Brooke, 1990; Dall'Antonia et al., 1995; Padget et al., 2022). By displacing shearwaters locally from their nest to nearby points around their home island's coastline, we provided shearwaters with a homing task where they had to choose between two over-water return routes. The absolute lengths of the two potential flight routes are small for a shearwater but differed more substantially in their relative cost. Thus, we examined how a long-distance traveller evaluates route choice at a fine spatial scale but out of direct sensory contact with the goal.

## MATERIALS AND METHODS

### Data collection and displacement procedures

This study took place on Skomer Island, Pembrokeshire, Wales, UK (51.737°N, 5.282°W), the site of the largest colony of Manx shearwaters, *Puffinus puffinus* (Brünnich, 1764), in the world (Perrins et al., 2019). Monitoring of nests throughout the season means that breeding stage and occupants of study nests were known prior to the experiment. Here, we targeted incubating (2023) or chick-rearing (2022 and 2023) adults of either sex, but which were of unknown age. Each individual was only displaced once per field season to minimise any cumulative impacts of repeated disturbance on their breeding attempt. Two birds displaced in 2022 were also displaced in 2023 (see Dataset 1).

Nests were monitored during the night for the arrival of adults, returning to their burrow to provision their chick or, in some cases in 2023 (11–16 May), to take over incubation from the partner, normally comprising a 5–7 day stint in the burrow (Brooke, 1990). For displacements during chick rearing (5–20 July), birds were removed from their burrows upon arrival, ideally before they began feeding their chick. The efficacy of displacements during chick rearing has been demonstrated in related species, apparently capitalising on birds remaining motivated to home when they have not yet fed their chick (Yoda et al., 2014). Similarly, previous work has shown that, during incubation, Manx shearwaters are highly motivated to home when displaced within the early stages of their incubation stints (Matthews, 1953; Padget et al., 2018). While still at the colony, 15 g Mobile Action i-gotU gt-120 GPS loggers were attached dorsally using thin strips of Tesa^®^ marine tape to small bunches of mantle feathers (see Dataset 1 for individual deployment masses), following a standard protocol outlined in Guilford et al. (2008). Devices were programmed to take fixes every second, providing high-resolution movement data, and were scheduled to begin recording after deployment.

**Fig. 1.**
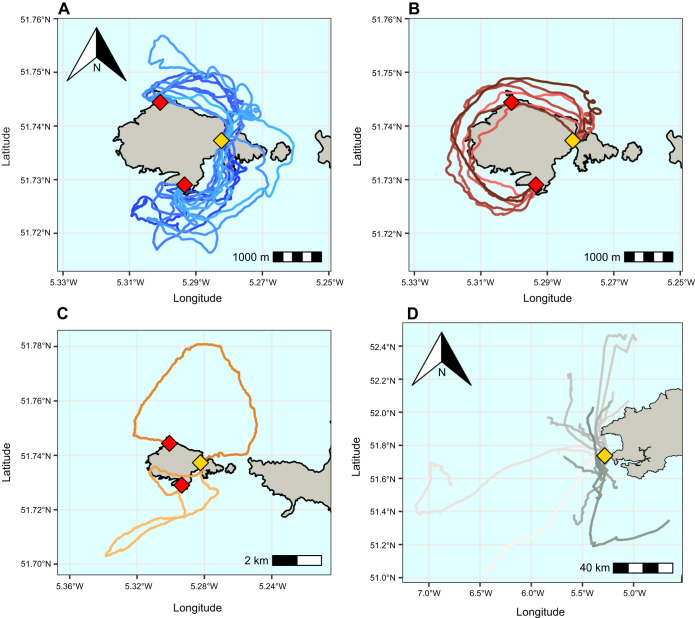
**Maps of the routes taken post-release by Manx shearwaters.** Routes are shown for birds that (A) homed immediately along the shorter route (*n*=20), (B) homed immediately along the longer route (*n*=6), (C) homed but showed non-classifiable behaviour (*n*=3) and (D) did not home upon release (*n*=20). The red points represent the two release sites, and the gold point indicates the colony location.

Birds were transported by foot (1.8–2.1 km) in opaque, sectioned boxes with disposable paper towel linings and air holes, to one of two release points around the coast of Skomer island (Fig. 1). Transport time did not differ between release sites and was consistent across individuals whilst total incarceration time (all time spent in a box, during tag attachment, transport and release) varied slightly between nights with the number of birds due to be released (see Dataset 1). As we anticipated that the shearwaters would be reluctant to fly over land directly back to the colony, the chosen release sites presented two over-sea routes back (one clockwise and one anticlockwise around Skomer Island) of unequal circumnavigation distances depending on the route adopted following release (Padget et al., 2019, 2022). From either site (Fig 1A,B), the shorter route option (eastwards) could be less than 2 km, whilst the longer route (initially westwards), would be around 5 km. In 2023, the presence of multiple handlers at the field site allowed for the simultaneous release of birds from both sites within one displacement night, maximising the number of birds that could be released on suitable nights. In 2022, a smaller field team meant that only one site could be visited during each night. Release site selection was pseudo-randomised to minimise any confounding effects.

Birds were released singly from atop 30 m high coastal cliffs from an open hand, allowing them to take flight immediately and ensuring a clear exit to sea. Handlers approximately directed each bird's take-off 180 deg from the homeward beeline at both sites to minimise any route biases induced by the release procedure. Each subsequent release was made only after the previous bird had vanished from sight. Wind speed thresholds of 7 m s^−1^, and gusts of 9 m s^−1^, were applied to any displacement night, to minimise both welfare risks and release night confounds.

Tag deployments and displacements were approved by the University of Oxford's Animal Welfare and Ethical Review Board in both 2022 and 2023 and approved by Natural Resources Wales, and the Islands Conservation Advisory Committee for Skomer and Skokholm Islands. GPS deployments and bird handling were under licence from the British Trust for Ornithology after review by the Special Methods Technical Panel (permit C/6981).

### Track processing

Only complete GPS tracks of birds that returned to the colony during the same night were used for analysis. These tracks were then filtered using speed thresholds for GPS errors (above 80 km h^−1^) and stationary bouts (0 km h^−1^, bird sat on the water) and trimmed with a 50 m exclusion zone around both the release site and nest site, to ensure that only homing flight behaviour (not take-off or landing) was analysed.

**Fig. 2.**
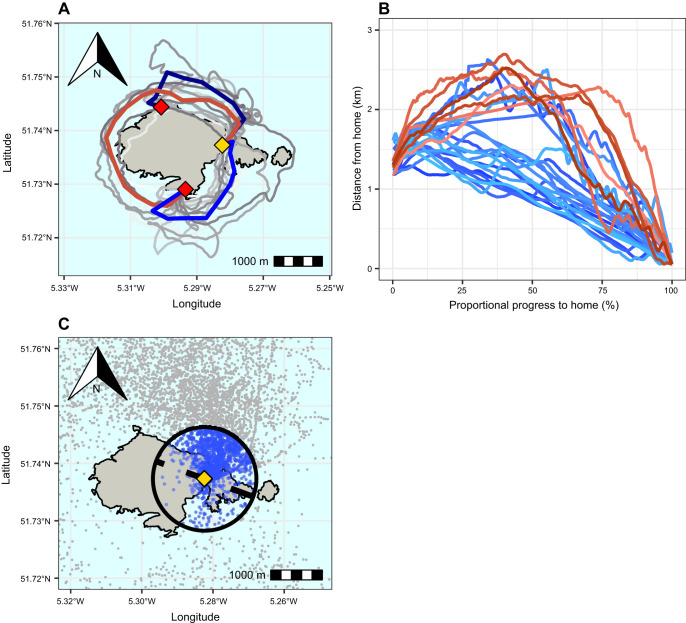
**Details of homing routes in displaced and free-ranging Manx shearwaters.** (A) A map showing all tracks from birds that homed immediately post-release in grey, overlayed with the median routes taken from each release site with respect to the shorter (blue) or longer (red) route taken. (B) The distance of each track from home as birds progress (measured as a proportion of their total flight distance) from release (0%) to return at the colony (100%), coloured as in A, where red tracks represent those that took the longer route and blue those that took the shorter. (C) A map showing the historical free-ranging tracking data collected from birds breeding on Skomer (*n*=887). Overlayed with a 1 km radius from the colony, the blue points showing fixes within this.

To visualise the routes shearwaters took back to their nest, a median route was calculated for each route (long and short) from each release site using data from all relevant birds. This was done by segmenting the island's coastline into radial polygons projecting from its centre, which encompassed all tracks, and then calculating the median latitude and longitude of all movement data within each of these polygon segments (Fig. 2A). For a polygon to contribute to the median route calculation, it must have contained tracks from at least three individuals, to avoid overrepresenting specific individuals.

### Route classification

Tracks were classified as ‘long’ or ‘short’ with respect to the route taken to circumnavigate the island following release. This was done by identifying the midpoint along each track and assigning it to either a clockwise (e.g. ‘short’ if released from the northern site) or anticlockwise (e.g. ‘long’ from the northern site) direction along the at-sea routes available post-release. Birds which flew either out to sea (*n*=2) or directly over the island (*n*=1) after release were excluded as the classification criteria above could not be applied (but are still presented in Fig. 1C).

### Environmental covariates

To test whether route choice was to minimise headwind encountered along their homing route, we modelled the effect of at-sea wind on route choice. Wind direction and speed were extracted from the European Centre for Medium-Range Weather Forecasts, Integrated Forecasting System (ECMWF IFS) dataset accessed through Open-Meteo (Hersbach et al., 2023; available from Zenodo: doi:10.5281/zenodo.7970649). The head/tailwind component that would have been experienced along the initial flight paths of both potential routes for each release time were calculated. These were included in models as positive values when the shorter route had a headwind and the longer route a tailwind and vice versa (Fig. 2A). Local weather measurements were not taken at release points, and the forecast data available did not have the spatial or temporal resolution to model individual wind conditions throughout the tracks, so values were taken from the closest time to release. Environmental variables related to ambient light (moon phase and cloud cover) were also modelled as night illumination is known to influence the night-flying behaviour of shearwaters (Siddiqi-Davies et al., 2024; Yamamoto et al., 2008).

### Analysis

Summary statistics (distance, mean speed and duration) were computed for each track, and each track was assigned a quadrant of the colony for its burrow position, as even this small difference in goal location (50–100 m) may well factor into route length calculations at this scale. Route preferences were tested against chance using two-tailed binomial tests and covariates included by using binomial linear regression models with long or short route as the response variable: Binary route choice∼Release site, family=binomial. Moon illumination and cloud cover were modelled together with an interaction term. Where covariates were categorical variables (e.g. release site), *P*-values were computed from an additional likelihood ratio test (LRT) between models with and without the predictor variable. Because some predictors created complete or quasi-complete separation, we applied Firth's penalised likelihood regression to obtain reliable coefficient estimates. Continuous route metrics were compared between the long and short routes using linear regression models.

To provide perspective on the birds' likely familiarity with the homing routes forced by displacement here, a long-term tracking dataset of free-ranging birds tracked from this colony was mapped (containing 887 foraging trips) and whether their final homing trajectory entered the colony from the northern or southern circumnavigation trajectory at 1 km distance from the colony was observed. This provided a route ratio of circumnavigations along either the north or south side of the island.

## RESULTS AND DISCUSSION

### Route choices in homing birds

Fifty-five birds were displaced to one of the two release sites (16 in 2022, all during chick rearing, and 39 in 2023, comprising 9 in incubation and 30 in chick rearing). All birds were subsequently re-encountered at the colony following displacement and all GPS devices were retrieved. The 55 releases resulted in 49 usable GPS tracks, in 29 of which birds homed immediately following release. Three homing routes were unclassifiable (Fig. 1C) and so were excluded from the analysis (but birds still ultimately made the ‘correct’ route choice from their respective release sites). Of the 26 homing trajectories analysed, 20 birds returned along the shorter of the circumnavigation routes, whilst 6 took the longer route, constituting a significant preference for the shorter route (two-tailed binomial test on 20/26, *P*=0.009; Fig. 1A,B).

None of release date (LRT χ²_8_=5.29, *P*=0.726), year (LRT χ²_1_=0.158, *P*=0.691), breeding stage (LRT χ²_1_=0.440, *P*=0.507), moon illumination (β=1.146, s.e.=1.739, *P*=0.510), cloud cover (β=−0.057, s.e.=0.040, *P*=0.156) and colony position of the target burrow (LRT χ²_1_=0.597, *P*=0.742) influenced route choice. Similarly, incarceration time did not influence the likelihood of birds homing (β=0.008, s.e.=0.035, *P*=0.826), or the route decisions of homing birds (β≤0.001, s.e.=0.057, *P*=0.994). Instantaneous headwind component (km h^−1^) along the shorter route did not predict the route choice (β=−0.001, s.e.=0.066, *P*=0.984). However, release site did marginally influence the odds of correct route decision: birds released from the southern site were more likely to adopt the longer route than those released from the northern site, from which all birds homed via the shorter route (LRT χ²_1_=3.97, *P*=0.046).

Choosing the shorter route was associated significantly with a lower total flight distance during homing (linear regression: *t*=2.575, *P*=0.017) and flight-only homing duration (*t=*2.645, *P*=0.014) (Table 1, Fig. 2B).

**Table 1.** Model outputs from linear regressions testing whether adopting the shorter or longer route influences homing trip metrics in Manx shearwaters

Metric	Decision	Mean±s.e.m.	*t*	*P*
Absolute homing duration (s)	Short	665±141	0.778	0.444
	Long	893±293		
Flight-only homing duration (s)	Short	405±64	2.575	0.017*
	Long	749±344		
Total flight distance to home (km)	Short	4.392±0.494	2.645	0.014*
	Long	7.109±1.027		
Flight speed (km h^−1^)	Short	42.058±2.291	−1.304	0.205
	Long	35.841±4.769		

Flight-only homing direction refers to the time spent in flight post-release, as displaced birds often sit on the water initially following release. Asterisks indicate significance.

### Island circumnavigation in natural tracks

The historic free-ranging shearwater GPS tracks revealed that 77.41% (677/887, *P*≤0.001, 95% confidence interval, CI: 73.36–79.06%) of all return trajectories from tracked foraging trips approached the colony from the north side of the island (Fig. 2C).

### Mechanisms of route preference

Here, we have demonstrated that shearwaters' navigational efficiency, well known at large scales, is also present at fine scales in situations where the potential benefit of adopting the efficient route is negligible. While the ability of shearwaters to find home from short distances after displacement is to be expected (as the final movements of larger-scale homing tasks are made across this scale: Gagliardo et al., 2013; Dall'Antonia et al., 1995; Padget et al., 2018), the current experiment nonetheless clearly shows the flexibility of whichever local navigational mechanism shearwaters use in the final stages of navigation home. This flexibility results in the successful choice of a marginally more efficient route home from either of two close-by locations. This efficient navigation following displacement, though simple, rules out reliance on the simplest navigational mechanisms known to guide organisms in their local familiar area, such as path integration (as here there is no outward journey information; Padget et al., 2019), beaconing towards home (as routes home offered here are indirect), or beaconing towards landmarks sequentially closer to home (steeple-chasing along a familiar route; Biro et al., 2004) which, while allowing homing, would do so inflexibly. While shearwaters incorporate wind conditions into foraging decisions at sea (Harris et al., 2025), wind direction and strength did not predict route choice here. If not a response to immediate wind conditions, these route choices imply that shearwaters knew which route was more efficient from both release sites.

General navigational strategies such as birds' ability to home from an unfamiliar site following displacement, which probably uses large-scale environmental gradients to compare a currently perceived location with those associated with home to chart a course between the two (true navigation), are unlikely to remain informative at such fine spatial scales (Lohmann and Lohmann, 2006; Mouritsen, 2018; Phillips, 1996; Wallraff, 2004, 2005). This is evidenced by the failure of shearwaters to anticipate island obstacles after natural homing, instead following an obstructed beeline towards home (Padget et al., 2019; Gagliardo et al., 2013). Successful anticipation of the shorter route here is therefore almost certainly reliant on birds' previous experience of the task (Griffin, 1952; Papi, 1990). Furthermore, the historical tracking data presented here show that the release site associated with more efficient route choices in our displacements (the northern site) is also encountered more often in natural movements and so is probably more familiar to Skomer Island shearwaters (Meade et al., 2005). Indeed, the inefficient choice of the longer route observed only from the southern site is reminiscent of familiar area route loyalties in pigeons where, despite more efficient routes being available, individuals will instead move to recapitulate routes with which they are more familiar, which here appears to be the northern side of the island (Biro et al., 2004; Meade et al., 2005). We did not design repeated releases into this experiment, but anecdotally a single individual (EM34657) successfully tracked in both years improved its choice from long to short from 2022 to 2023, and it is entirely plausible that increased experience obtained between the years facilitated this switch.

Over land, homing pigeons repeatedly released from beyond topographical obstacles progressively improve their performance with the navigational task, probably through learning and route refinement (Bonadonna et al., 1997). At sea, Scopoli's shearwaters (*Calonectris diomedea*) displaced beyond Mediterranean islands generally make efficient circumnavigation choices upon homing (Sicily: Dall'Antonia et al., 1995; Corsica: Pollonara et al., 2015), demonstrating route knowledge within a broad familiar area (Pollonara et al., 2015). At these scales (tens to hundreds of kilometres) it is clear how such routes could be learnt associatively, as differences in the costs of alternative route choices would plausibly be salient to the birds (Bonadonna et al., 1997; Dall'Antonia et al., 1995; Guilford and Burt de Perera, 2017; Pearce, 2008). Similar learning might underpin the route efficiencies reported here, perhaps achieved through repeated experience arriving at the home island from different directions during natural movements. However, here, the pay-off between route choices was small, and so the different underlying learning mechanisms potentially operating at this fine scale deserve careful consideration (Kacelnik et al., 2023), even if we cannot resolve them fully at this stage.

First, we consider whether these routes could be learnt through generalisable associative learning during previous natural round-island movements, where the reward of reaching home sooner would reinforce the route choice on future occasions (Guilford and Burt de Perera, 2017; Pearce, 2008). For this association to form, however, the difference in the perceived value between the two options must be detectable (Guilford and Burt de Perera, 2017). In classical choice experiments, this value would be apparent in the prescribed magnitude of the reward received (Sutherland and Mackintosh, 1971), which here would translate to the reduction in flight time taken to reach the reward of arriving back at the nest. Whilst these routes indeed differ in such value, the detectability of this difference is not a given, as at this spatial scale the temporal costs of these routes are near identical in absolute terms (a difference between mean flight timings of just 344 s), particularly when considering the typical distances travelled by this species (Guilford et al., 2008; Padget et al., 2019). The similarity in route values may therefore impede generalised associative learning.

Despite the absolute difference in route cost being small, the shorter routes still offer a near 50% reduction in distance and subsequent flight time to reach the colony (Table 1). In cognitive science, Weber's Law states that proportional contrast is of greater importance than absolute contrast in the salience of a dichotomy (Brus et al., 2019). This law could be invoked at this fine scale to explain how routes which offer high relative, but low absolute, differences in reward could still be learnt under a generalised associative framework, if birds are capable of such a relative comparison (Bateson and Kacelnik, 1995).

Alternatively, if these small-scale route efficiencies are not underpinned by generalised associative learning, then some kind of ‘latent learning’ may be involved. Latent learning is broadly defined as the association of stimuli without direct reinforcement and employs a specially evolved cognitive module to pay attention to, and associate, primed stimuli without the apparent rewards required under an associative framework (Wang and Hayden, 2021). These modules have evolved to recognise specific stimuli, usually of significant fitness importance to the animal (Öhman and Mineka, 2001), at the cost of the generality of associative learning, where any salient stimulus can be learnt. In the context of navigation, this led to the expectation of a module evolved for the detection of route efficiencies and the subsequent discovery of the cognitive map, which appears to facilitate learning and recall of efficient routes (and other spatial information) in the absence of reward in a range of taxa (Singer et al., 2006; Toledo et al., 2020; Tolman, 1948; Wang and Hayden, 2021), and could be involved here too. Whilst concluding which learning mechanism may operate at these fine scales is beyond the inference of these results, such questions as to how the route flexibility demonstrated here is obtained offer new avenues of investigation into the cognitive underpinnings of navigation within this species.

Here, we have shown that shearwaters are able to make efficient route choices, even at fine scales, and that this is unlikely to be underpinned by the generalised navigation strategies employed when homing over longer distances. Instead, familiarity with the task and the learning of optimal routes probably facilitates this. This learning could be either latent, such as the cognitive map, or associative, if shearwaters are able to detect such nuanced differences in route rewards. We therefore suggest that future studies should aim to manipulate the rewards of getting home (for example by delaying the entry to the nest for specific route choices) to switch their route preferences to gain further insight into the detection and learning of these small-scale route efficiencies.

## Supplementary Material

10.1242/jexbio.251556_sup1 Supplementary information

Supplementary Materials. A dataset containing all metadata and environmental data for each bird displaced within this experiment. Transport time is calculated as the time taken to walk from the colony to the release site, whilst total incarceration time is the cumulative duration the bird spent in a holding box throughout GPS deployment, transport, and release.
