# Supplementary material for: Shearwaters make efficient navigational decisions, even at very fine scales
Source: J Exp Biol. 2026 Apr 30;229(9):jeb251556. doi: 10.1242/jeb.251556 (PMC13200727; doi:10.1242/jeb.251556)
Supplement: Supplementary information [file jexbio-229-251556-s1.pdf]

Supplementary Materials. A dataset containing all metadata and environmental data for each bird displaced within this experiment. Transport time is calculated as the time taken to walk from the colony to the release site, whilst total incarceration time is the cumulative duration the bird spent in a holding box throughout GPS deployment, transport, and release.

Available for download at
https://journals.biologists.com/jeb/article-lookup/doi/10.1242/jeb.251556#supplementary-data
